# Self-Assembling Hybrid Linear-Dendritic Block Copolymers: The Design of Nano-Carriers for Lipophilic Antitumoral Drugs

**DOI:** 10.3390/nano9020161

**Published:** 2019-01-29

**Authors:** Elisabetta Fedeli, Alexandre Lancelot, Juan Manuel Dominguez, José Luis Serrano, Pilar Calvo, Teresa Sierra

**Affiliations:** 1 Departamento de Química Orgánica, Instituto de Nanociencia de Aragón (INA), Instituto de Ciencia de Materiales de Aragón (ICMA), Edificio I+D, Universidad de Zaragoza, 50018 Zaragoza, Spain; elife83@gmail.com (E.F.); alexandrelancelot@gmail.com (A.L.); joseluis@unizar.es (J.L.S.); 2 PharmaMar S.A., 28770 Colmenar Viejo, Madrid, Spain; jmdominguez@pharmamar.com; 3 Departamento de Química Orgánica, Instituto de Ciencia de Materiales de Aragón (ICMA), Facultad de Ciencias, CSIC, Universidad de Zaragoza, 50009 Zaragoza, Spain

**Keywords:** dendrimers, micellar aggregates, drug delivery, anticancer

## Abstract

Two series of amphiphilic block copolymers with a hybrid linear-dendritic structure are presented. The compounds consisted of a hydrophilic poly (ethylene glycol) (PEG) block and a 2,2′-bis(hydroxymethyl)propionic acid (bis-MPA) dendron functionalized with stearic acid chains that impart a hydrophobic nature to the block. Different self-assembled nanostructures with a hydrophobic interior and a hydrophilic external part were obtained depending on the length of the PEG chain (*M*_n_ = 2000 and *M*_n_ = 5000) and the generation of the bis-MPA dendron. The materials were characterized by transmission electron microscopy (TEM). The shapes of the aggregates ranged from spherical or cylindrical micelles to flexible bilayers. The hydrophobic core enabled these nanostructures to encapsulate the water-insoluble drug plitidepsin. The efficacy of these new plitidepsin-containing carriers was evaluated in four cancer cell-lines and they showed similar anticancer activity to the current standard drug formulation.

## 1. Introduction

Polymer-based carriers for anticancer drugs bring significant benefits to cancer therapy and these include responsiveness to external stimuli, increased drug efficacy, localized drug delivery, and decreased side effects [1,2,3]. Among the different polymeric architectures, hybrid linear-dendritic block copolymers are perfectly suited for several applications in the biomedical field, including the encapsulation of guest molecules, the delivery of genetic material, and the ability to mimic the cell membrane [4,5,6,7,8].

Hybrid linear-dendritic block copolymers (HLDBCs) are macromolecules composed of a linear polymeric chain linked at one end with a dendrimeric wedge. These materials contain segments of different molecular architectures and combine the characteristics of the rigid, globular, multi-branched dendritic wedge with the high mobility of the linear polymeric chain [9,10]. The appropriate choice of the blocks and the possibility of functionalizing ad hoc the surface of the dendrons allow the design of amphiphilic macromolecules that can form complex supramolecular architectures [11,12,13,14]. Amphiphilic block copolymers differ from low molecular weight surfactants in that they allow a better control of the self-assembly process, as determined both by the chemical nature and the length of the polymer blocks. In addition, the presence of a more rigid dendritic part seems to stabilize further the supramolecular structures by decreasing the mobility of the polymeric chain and improving the properties of the aggregates in vitro and in vivo [9,15,16].

Poly(ethylene glycol) (PEG) polymers and 2,2′-bis(hydroxymethyl)propionic acid (bis-MPA) based dendrimers are widely employed in biocompatible HLDBCs. The use of PEG in biomedical applications began in the early 90s as a coating polymer to protect liposomes from the mononuclear phagocytic system (MPS) [17,18]. The mechanism that prevents the recognition of PEG-coated liposomes by the MPS is not fully understood, but the widely accepted hypothesis is that the PEG coating reduces the interactions between particles and inhibits the adsorption of various opsonin (immune and non-immune serum factors that are able to enhance the phagocytosis process), thus increasing the biological stability and the circulation time [19]. Bis-MPA-based dendrimers are easy to synthesize by convergent or divergent methods and they have proven to be ideal for the construction of drug carriers due to their low cytotoxicity and high biodegradability [20,21,22].

**Figure 1:**
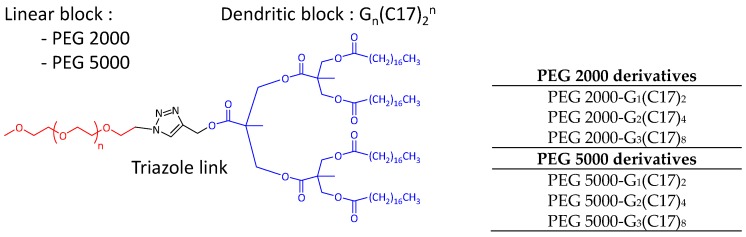
Representative chemical structure of the amphiphilic hybrid linear-dendritic block copolymers (PEG-G_2_(C17)_4_ is shown as a representative example) and list of all synthesized compounds. The molecular weight of the linear polyethylene glycol (PEG 2000 *M*_n_ or 5000 *M*_n_) is included in the name of the molecule, followed by the generation of the dendritic block and the corresponding number of alkyl chains.

We report here a series of amphiphilic hybrid linear-dendritic block copolymers (HLDBCs) in which the hydrophilic polymeric block is composed of PEG and the lipophilic dendritic wedge is based on bis-MPA. In an effort to modulate the amphiphilic properties of the HLDBCs, six compounds were synthesized by combining poly(ethylene glycol) methyl ether azides of two different molecular weights (5000 *M*_n_ and 2000 *M*_n_) with the first-, second-, and third-generation lipophilic bis-MPA dendrons (Figure 1). Different sizes of PEG blocks and different generations of dendrons were chosen in order to tune the amphiphilic nature of the final HLDBCs and thus the influence on self-aggregation and drug encapsulation. The structures were designed to self-assemble in water to give supramolecular aggregates, which can be useful as nano-carriers for the encapsulation and delivery of lipophilic molecules [23,24] such as plitidepsin, an anticancer drug discovered by PharmaMar [25,26].

## 2. Materials and Methods

### 2.1. Materials

Poly(ethylene glycol)methyl ether azide (*M*_n_ 2000 and *M*_n_ 5000), 2,2′-bis(hydroxymethyl)propionic acid (bis-MPA), stearic acid, Pd/C, CuSO_4_, sodium ascorbate, KCN, propargyl alcohol, DOWEX^®^ ion exchange resin, *N*,*N*′-dicyclohexylcarbodiimide (DCC), 4-dimethylaminopyridine (DMAP), and 4-(dimethylamino)pyridinium *p*-toluenesulfonate (DPTS) were purchased from Aldrich and were used without further purification. Magnesium sulfate, CH_2_Cl_2_, methanol, dimethylformamide (DMF), hexane, and ethyl acetate were purchased from Scharlab. CH_2_Cl_2_ was dried using a solvent purification system before use. Plitidepsin was supplied by PharmaMar and was used without further purification. Ultrapure Milli-Q water (ρ = 18.2 MΩ∙cm^−1^) was used to induce the formation of aggregates.

### 2.2. Synthesis of the Materials

The synthesis of the alkyne-functionalized dendritic blocks has been described previously [27]. General method for the Cu(I)-catalyzed alkyne-azide cycloaddition (CuAAC): 0.5 mol of CuSO_4_ and 1 mol of sodium ascorbate were dissolved in 2 mL of a mixture of CH_2_Cl_2_:DMF:H_2_O (3:1:1) in a vial, and the mixture was stirred for 1 h at room temperature. 1 mol of alkyne-functionalized dendron and 1 mol of azide-functionalized PEG monomethyl ether derivative, dissolved in 5 mL of CH_2_Cl_2_, was added to the solution of CuSO_4_ and sodium ascorbate. The reaction mixture was stirred for 48 h at 40 °C. The reaction mixtures were washed with aqueous KCN solution to remove residual traces of copper. The organic phase was separated and dried over magnesium sulfate. The solvent was evaporated under reduced pressure. Further purifications were performed depending on the specific case.

**PEG2000-G_1_(C17)_2_.** The crude product was dissolved in a small amount of dichloromethane and was precipitated with cold hexane. The solid was filtered off and washed several times, first with hexane and then with methanol. Yield: 56%, colorless solid. ^1^H-NMR (400 MHz, CDCl_3_, δ): 7.77 (s, 1H, –N–CH–C); 5.24 (s, 2H, –N–C–CH_2_–OCO); 4.54 (t, 2H, –O–CH_2_–CH_2_–N); 4.20 (ABq, 4H, *J* = 11 Hz, Δν_AB_ = 16.7 Hz, C–CHH′–); 3.87 (t, 2H, –O–CH_2_–CH_2_–N); 3.75–3.52 (m, ≈176H, – (CH_2_–CH_2_)_44_–); 3.37 (s, 3H, –(CH_2_–CH_2_)_44_–O–CH_3_); 2.24 (t, 4H, –OCO–CH_2_–CH_2_–(CH_2_)_14_–CH_3_); 1.56 (m, 4H, –OCO–CH_2_–CH_2_–(CH_2_)_14_–CH_3_); 1.29–1.22 (m, 56H, –OCO–CH_2_–CH_2_–(CH_2_)_14_–CH_3_); 1.21 (s, 3H, C–CH_3_); and 0.87 (t, 6H, –OCO–CH_2_–CH_2_–(CH_2_)_14_–CH_3_). ^13^C-NMR (100 MHz, CDCl_3_, δ): 110.1, 77.4, 72.1, 70.7, 59.2, 34.2, 32.1, 29.9, 29.6, 29.5, 29.4, 29.3, 22.8, and 14.26. FTIR (cm^−1^, Nujol): 2941–2848 (ν, C–H); 1710 (ν, C=O); and 1459 (δ –CH_2_–; δ_as_ –CH_3_). *M*_n_ (MALDI): 2317 Ð: 1.04.

**PEG2000-G_2_(C17)_4_.** The crude product was dissolved in a small amount of dichloromethane and was precipitated with cold hexane. The solid was filtered off and washed several times, first with hexane and then with methanol. The starting materials were removed by dialysis against dichloromethane for one day with a Millipore membrane with a 1000 MW cut-off. Yield: 50%, colorless solid. ^1^H-NMR (400 MHz, CDCl_3_, δ): 7.80 (s, 1H, –N–CH–C); 5.24 (s, 2H, –N–C–CH_2_–OCO); 4.55 (t, 2H, –O–CH_2_–CH_2_–N); 4.23 (ABq, 4H, *J* = 11 Hz, Δν_AB_ = 9.4 Hz, C–CHH′– first generation); 4.14 (m, 8H, C–CHH′– second generation); 3.89 (t, 2H, –O–CH_2_–CH_2_–N); 3.69–3.53 (m, ≈176H, –(CH_2_–CH_2_)_44_–); 3.37 (s, 3H, –(CH_2_–CH_2_)_44_–O–CH_3_); 2.28 (t, 8H, –OCO–CH_2_–CH_2_–(CH_2_)_14_–CH_3_); 1.57 (m, 8H, –OCO–CH_2_–CH_2_–(CH_2_)_14_–CH_3_); 1.31–1.23 (m, 112H, –OCO–CH_2_–CH_2_–(CH_2_)_14_–CH_3_); 1.22 (s, 3H C–CH_3_ first generation); 1.18 (s, 6H, C–CH_3_ second generation); and 0.87 (t, 12H, –OCO–CH_2_–CH_2_–(CH_2_)_14_–CH_3_). ^13^C-NMR (100 MHz, CDCl_3_, δ): 173.2, 172.12, 172.06, 141.9, 125.3, 110.1, 72.0, 71.0, 70.8, 70.6, 70.1, 69.4, 65.5, 65.0, 59.1, 58.4, 50.7, 50.3, 46.7, 46.4, 32.0, 29.8, 29.7, 29.6, 29.4, 29.4, 29.2, 24.9, 22.8, and 14.2. FTIR (cm^−1^, Nujol): 2942–2848 (ν, C–H); 1733 (ν, C=O); and 1465 (δ –CH–; δ_as_ –CH_3_). *M*_n_ (MALDI): 3324 Ð: 1.00.

**PEG2000-G_3_(C17)_8_.** The crude product was dissolved in a small amount of dichloromethane and was precipitated with cold hexane. The solid was filtered off and washed several times, first with hexane and then with methanol. The starting materials were removed by dialysis against dichloromethane for one day with a Millipore membrane with a 1000 MW cut-off. The resulting product was dissolved in a small amount of dichloromethane and was precipitated with cold methanol. The colorless solid was washed with hot hexane. The precipitation in methanol and washing with hexane were repeated several times in order to purify the product. Yield: 48%, colorless solid. ^1^H-NMR (400 MHz, CDCl_3_, δ): 7.81 (s, 1H, –N–CH–C); 5.24 (s, 2H, –N–C–CH_2_–OCO); 4.54 (t, 2H, –O–CH–CH_2_–N); 4.27–4.13 (m, 28H, C–CHH′– first, second, and third generations); 3.88 (t, 2H, –O–CH_2_–CH_2_–N); 3.73–3.52 (m, ≈176H, –(CH_2_–CH_2_)_44_–); 3.37 (s, 3H, –(CH_2_–CH_2_)_44_–O–CH_3_); 2.28 (t, 16H, –OCO–CH_2_–CH_2_– (CH_2_)_14_–CH_3_); 1.57 (m, 16H, –OCO–CH_2_–CH_2_–(CH_2_)_14_–CH_3_); 1.31–1.22 (m, 227H, –OCO–CH_2_–CH_2_–(CH_2_)_14_–CH_3_; C–CH_3_ first generation); 1.21 (s, 12H, C–CH_3_ third generation); 1.18 (s, 6H, C–CH_3_ second generation); and 0.87 (t, 24H, –OCO–CH_2_–CH_2_–(CH_2_)_14_–CH_3_). ^13^C-NMR (100 MHz, CDCl_3_, δ): 173.3, 172.2, 72.1, 70.7, 65.0, 57.8, 46.5, 34.2, 32.1, 29.9, 29.8, 29.7, 29.5, 29.4, 29.3, 25.1, 22.8, 17.9, and 14.3. FTIR (cm^−1^, Nujol): 2942–2848 (ν, C–H); 1735 (ν, C=O); and 1463 (δ –CH_2_–; δ_as_ –CH_3_). *M*_n_ (MALDI): 4902 Ð: 1.05.

**PEG5000-G_1_(C17)_2_.** The crude product was dissolved in a small amount of dichloromethane and was precipitated with cold hexane. The solid was filtered off and washed several times, first with hexane and then with methanol. Yield: 62%, colorless solid. ^1^H-NMR (400 MHz, CDCl_3_, δ): 7.76 (s, 1H, –N–CH–C); 5.25 (s, 2H, –N–C–CH_2_–OCO); 4.54 (t, 2H, –O–CH_2_–CH_2_–N); 4.20 (ABq, 4H, *J* = 11 Hz, Δν_AB_ = 16.7 Hz, C–CHH′–); 3.87 (t, 2H, –O–CH_2_–CH_2_–N); 3.73–3.53 (m, ≈448H, –(CH_2_–CH_2_)_112_–); 3.38 (s, 3H, –(CH_2_–CH_2_)_112_–O–CH_3_); 2.25 (t, 4H, –OCO–CH_2_–CH_2_–(CH_2_)_14_–CH_3_); 1.56 (m, 4H, –OCO–CH_2_–CH_2_–(CH_2_)_14_–CH_3_); 1.31–1.22 (m, ≈56H, –OCO–CH_2_–CH_2_–(CH_2_)_14_–CH_3_); 1.21 (s, 3H, C–CH_3_); and 0.87 (t, 6H, –OCO–CH_2_–CH_2_–(CH_2_)_14_–CH_3_). ^13^C-NMR (100 MHz, CDCl_3_, δ): 111.4, 77.4, 70.7, 59.2, 34.2, 32.1, 29.8, 29.8, 29.6, 26.5, 29.4, 29.3, 25.0, 22.80, and 14.3. FTIR (cm^−1^, Nujol): 2941–2848 (ν, C–H); 1712 (ν, C=O); and 1461 (δ –CH_2_–; δ_as_ –CH_3_). *M*_n_ (MALDI): 5399 Ð: 1.05.

**PEG5000-G_2_(C17)_4_.** The crude product was dissolved in a small amount of dichloromethane and was precipitated with cold hexane. The solid was filtered off and washed several times, first with hexane and then with methanol. The starting materials were removed completely by dialysis of the product against dichloromethane for one day with a Millipore membrane with a 1000 MW cut-off. Yield: 70%, colorless solid. ^1^H-NMR (400 MHz, CDCl_3_, δ): 7.80 (s, 1H, –N–CH–C); 5.25 (s, 2H, –N–C–CH_2_–OCO); 4.55 (t, 2H, –O–CH_2_–CH_2_–N); 4.23 (ABq, 4H, *J* = 11Hz, Δν_AB_ = 9.4, C–CHH′– first generation); 4.13 (m, 8H, C–CHH′– second generation); 3.89 (t, 2H, –O–CH_2_–CH_2_–N); 3.72–3.52 (m, ≈444H, –(CH_2_–CH_2_)_112_–); 3.37 (s, 3H, –(CH_2_–CH_2_)_112_–O–CH_3_); 2.28 (t, 8H, –OCO–CH_2_–CH_2_– (CH_2_)_14_–CH_3_); 1.57 (m, 8H, –OCO–CH_2_–CH_2_–(CH_2_)_14_–CH_3_); 1.31–1.19 (m, 112H, –OCO–CH_2_–CH_2_– (CH_2_)_14_–CH_3_); 1.22 (s, 3H, C–CH_3_ first generation); 1.17 (s, 6H, C–CH_3_ second generation); and 0.87 (t, 12H, –OCO–CH_2_–CH_2_–(CH_2_)_14_–CH_3_). ^13^C-NMR (100 MHz, CDCl_3_, δ): 173.3, 172.1, 125.3, 110.1, 72.0, 71.1, 70.7, 69.5, 65.5, 65.1, 59.1, 58.5, 50.3, 46.7, 46.4, 32.0, 29.8, 29.7, 29.6, 29.5, 29.4, 29.2, 25.0, 22.8, 17.9, and 14.2. FTIR (cm^−1^, Nujol): 2942–2848 (ν, C–H); 1731 (ν, C=O); and 1465 (δ –CH_2_–; δ_as_ –CH_3_). *M*_n_ (MALDI): 6202 Ð: 1.04.

**PEG5000-G_3_(C17)_8_.** The crude product was dissolved in a small amount of dichloromethane and was precipitated with cold methanol. The colorless solid was filtered off and washed with methanol and hexane several times. Yield: 63%, colorless solid. ^1^H-NMR (400 MHz, CDCl_3_, δ): 7.80 (s, 1H, –N–CS–C); 5.24 (s, 2H, –N–C–CH_2_–OCO); 4.54 (t, 2H, –O–CH_2_–CH_2_–N); 4.24–4.13 (m, 28H, C–CHH′– first, second, and third generation); 3.89 (t, 2H, –O–CH_2_–CH_2_–N); 3.69–3.51 (m, ≈448H, – (CH_2_–CH_2_)_112_–); 3.37 (s, 3H, –(CH_2_–CH_2_)_112_–O–CH_3_); 2.28 (t, 16H, –OCO–CH_2_–CH_2_–(CH_2_)_14_–CH_3_); 1.57 (m, 16H, –OCO–CH_2_–CH_2_–(CH_2_)_14_–CH_3_); 1.31–1.22 (m, 227H, –OCO–CH_2_–CH_2_–(CH_2_)_14_–CH_3_; C–CH_3_ first generation); 1.21 (s, 12H, C–CH_3_ third generation); 1.18 (s, 6H, C–CH_3_ second generation); and 0.87 (t, 24H, –OCO–CH_2_–CH_2_–(CH_2_)_14_–CH_3_). ^13^C-NMR (100 MHz, CDCl_3_, δ): 173.3, 172.2, 110.1, 77.4, 72.1, 70.7, 65.0, 59.2, 46.8, 46.5, 34.2, 32.1, 29.85, 29.8, 29.7, 29.5, 29.3, 25.0, 22.8, 17.9, and 14.3. FTIR (cm^−1^, Nujol): 2941–2846 (ν, C–H); 1731 (ν, C=O); and 1465 (δ –CH_2_–; δ_as_ –CH_3_). *M*_n_ (MALDI): 7830 Ð: 1.08.

### 2.3. Formation of the Aggregates and Morphological Studies

The formation of aggregates was studied by evaluating the critical micelle concentration (CMC) of the HLDBCs in water. The formation of aggregates was achieved by the oil-in-water method, in which each compound was dissolved in a volatile solvent (0.5 mM in dichloromethane) and then Milli-Q water was added and the mixture was stirred until complete evaporation of the organic fraction [28]. The amount of amphiphile corresponded to a final concentration in water of 0.5 mM. The resulting aggregates were studied by transmission electron microscopy, TEM (TECNAI G20, 200 kV), using 0.1 N uranyl acetate as negative stain. TEM samples were prepared by placing a drop of aggregate solution on a TEM grid (holey carbon film on 300 mesh copper, Agar Scientific, (Essex, CM24 8GF, UK). Water from the sample was removed by capillarity with a filter paper. The sample was dried overnight in the dark.

### 2.4. Plitidepsin Encapsulation

The ability of the aggregates to encapsulate a lipophilic guest was evaluated by the oil-in-water method, which was specifically modified for this procedure [29]. The amphiphile and plitidepsin were dissolved in dichloromethane. The amount of each component corresponded to a final concentration of the amphiphile in water of 0.5 mM and 1 mM (1.11 mg/mL) of plitidepsin. This concentration of the drug was selected in order to exploit fully the payload capacity of the aggregates (see Supplementary Materials Section S3).

### 2.5. Cytotoxicity Studies

The cytotoxicity levels of the HLDBCs and their plitidepsin-containing host–guest complexes were evaluated at the PharmaMar laboratories in four human cancer cell-lines purchased from the ATCC: A549 (lung, non-small cell lung cancer), HT29 (colon), MDA-MB-231 (breast), and PSN1 (pancreas). Assays were performed in 96-well plates with 5000 cells per well in a final volume of 200 µL of Dulbecco’s modified eagle medium (DMEM) supplemented with 10% fetal bovine serum (FBS), 2mM l-glutamine, 100 U/mL penicillin, and 100 U/mL streptomycin. Compounds were tested at several concentrations in a range covering 10 serial 2/5 dilutions starting from 1 µg/mL (from 0.1 µg/mL for plitidepsin), i.e., 1, 0.4, 0.16, 0.064, 0.0256, 0.01024, 0.004096, 0.001638, 0.000655, and 0.000262 µg/mL (10-fold lower for plitidepsin) in 1% (*v*/*v*) DMSO. Cells and compounds were incubated for 72 h at 37 °C in 5% CO_2_ and 98% humidity, and cell growth and viability were subsequently measured by a colorimetric procedure with sulforhodamine B as described in the literature [30]. All evaluations were performed in triplicate. Three reference parameters that defined the effect of the compound were calculated: GI50 (compound concentration that produced 50% cell growth inhibition when compared to control cultures); TGI (compound concentration that caused total cell growth inhibition, i.e., a cytostatic effect, when compared to control cultures); and LC50 (compound concentration that produced a 50% net cell death cytotoxic effect).

### 2.6. Statistical Analysis

The data obtained from the cytotoxicity studies were fitted by non-linear regression to a four-parameter logistic curve with the algorithm developed by the American National Cancer Institute (ANCI) [31].

## 3. Results and Discussion

### 3.1. Synthesis and Characterization

**Scheme 1:**
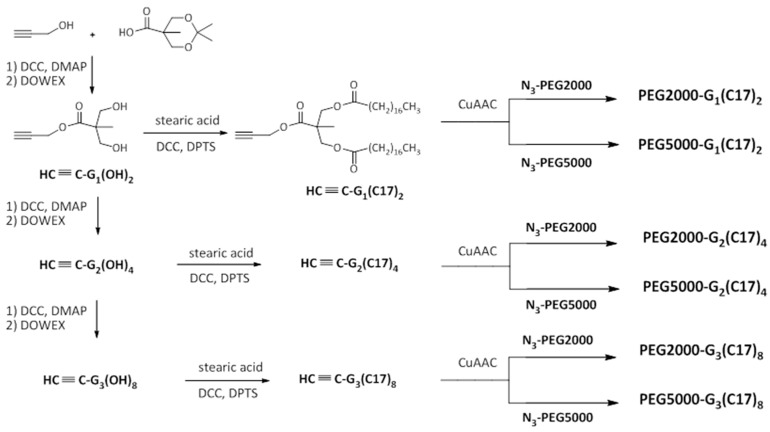
Synthetic route for the hybrid linear-dendritic block copolymers (HLDBCs).

The synthesis of the HLDBCs is outlined in Scheme 1. The synthesis of the dendritic blocks was previously described by us [27]. Briefly, the first step involved the esterification of the acetonide-protected bis-MPA monomer with propargyl alcohol. Subsequent deprotection of the terminal –OH groups with DOWEX^®^ ion exchange resin provided HC≡C–G_1_(OH)_2_. The dendron generation was grown by the Steglich procedure [32], in which DCC is used along with DPTS or DMAP as activating agents. The periphery of all three dendrons was functionalized by esterification with stearic acid.

The last step of the synthesis was the coupling of each dendritic block (HC≡C–G_n_(C17)_m_) with commercially available azide-terminated linear hydrophilic PEGs (either PEG2000-N_3_ or PEG5000-N_3_) by Cu(I)-catalyzed azide/alkyne cycloaddition (CuAAC). The reaction was performed with CuSO_4_·5H_2_O and sodium ascorbate as the catalytic system.

**Figure 2:**
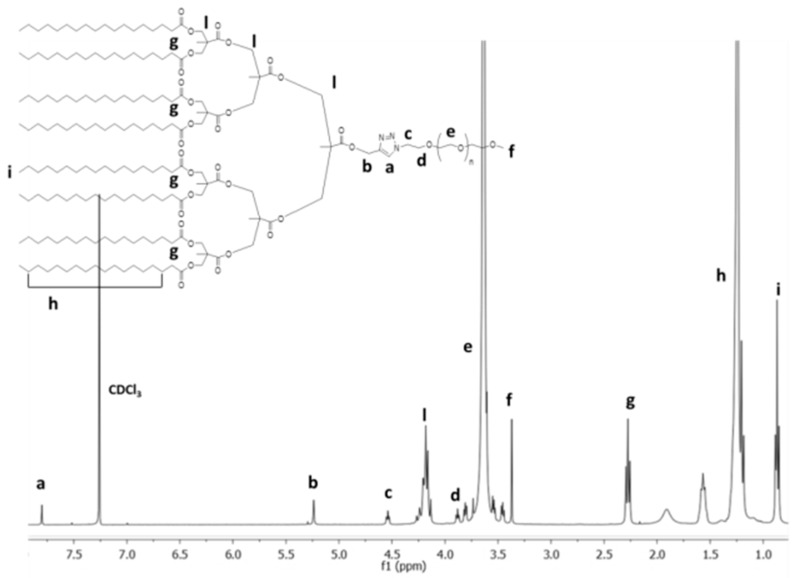
^1^H-NMR spectrum in CDCl_3_ of the HLDBC PEG5000-G_3_(C17)_8_.

The success of the CuAAC reaction was assessed by FTIR and ^1^H NMR spectroscopy. The FTIR spectrum of the HLDBC clearly showed the disappearance of the characteristic peaks of the starting materials, i.e., the azide group (2100 cm^−1^) of the poly(ethylene glycol)methyl ether azide and the peak corresponding to the stretching vibration of the H–C bond of the alkyne group (3300 cm^−1^) of the dendritic block (Figure S2). The ^1^H NMR spectrum was consistent with the formation of the triazole ring, as evidenced by the new signal for proton **a** (δ_H_ = 7.80 ppm, Figure 2) of the –CH group of the newly formed heterocycle. Gel permeation chromatograms showed monomodal peaks for all compounds and these had lower retention times than the corresponding starting materials; this indicated the absence of residues due to starting materials (Figure S3). MALDI-TOF mass spectrometry highlighted the shift of the peak distribution typical of PEG to higher *m*/*z* values, which corresponded to an increase in the molecular weight of the final compounds with respect to the starting materials (Figure S4).

### 3.2. Self-Assembly in Water

Prior to studying the formation of aggregates in water, the concentration at which the amphiphilic molecules started to self-assemble into micelles, i.e., the critical micelle concentration (CMC) [33,34,35], was determined.

The CMC values of all six HLDBCs are determined by the pyrene fluorescence method [36], and are provided in Table 1. The CMC values were consistent with the data generally reported for amphiphilic block copolymers (10^−5^–10^−7^ M) [37,38]. The variation of the dimensions of the blocks (length of PEG or generation of the dendritic block) did not seem to affect the CMC value significantly, and this was of the same order of magnitude in all cases, i.e., within the range 1.4–3.1·10^−6^ M. All of the compounds showed good solubility in water, with values of at least ca. 0.5 mM—the concentration at which the aggregation and encapsulation experiments were performed.

**Figure 3:**
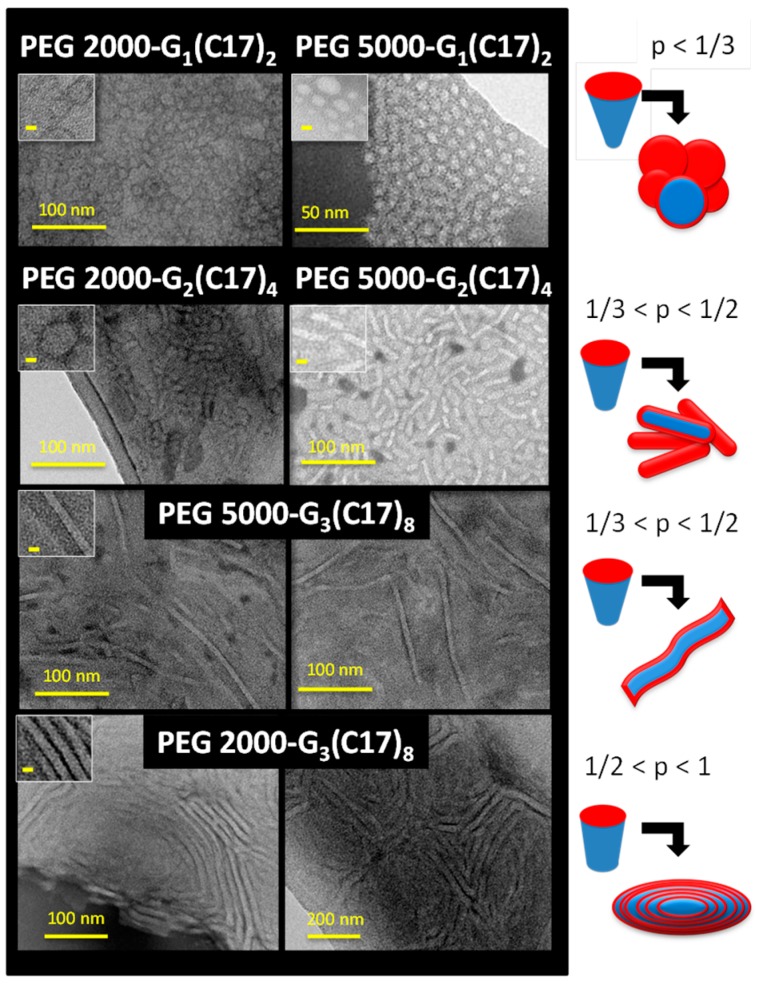
(**Left**) TEM images of the six HLDBCs. Magnified images of the aggregates are displayed in the insets; the yellow scale bar corresponds to 10 nm. (**Right**) For comparison, schematic representation of the different morphologies predicted by the packing parameter models [39].

The architectures formed by the HLDBCs in water after self-assembly were studied by TEM microscopy (Figure 3, left). The differences between the architectures can be related to the sizes of the two different parts—the linear hydrophilic part and the dendritic lipophilic part. Based on the morphologies observed in the TEM images, an empirical classification of the HDLBCs within the packing parameter ranges described for surfactants was proposed (Figure 3, right) [39]. The packing parameter (*p*) is a value that represents the ratio between the volume of the lipophilic part (stearic chains) of a surfactant and its hydrophilic surface, and this parameter correlates to the aggregate architecture [40,41].

Comparison of the pairs of compounds that contain the same generation of dendritic block and the two different molecular weights of PEG chain indicated that marked differences were not apparent between the shapes of the aggregates. Compounds PEG 2000-G_1_(C17)_2_ and PEG 5000-G_1_(C17)_2_ had a low lipophilic content when compared to the hydrophilic part, and this led to the formation of spherical aggregates. This finding was consistent with the theoretical model proposed for amphiphiles with a packing parameter of less than 1/3, which led to the formation of spherical micelles. Hence, these aggregates were interpreted as spherical micelles with a mean diameter of ≈10 nm.

In contrast to the first-generation HLDBCs, elongated micelles with mean short axes of ca. 10 nm were observed in TEM images corresponding to PEG 2000-G_2_(C17)_4_ and PEG 5000-G_2_(C17)_4_ (Figure 3), probably due to the increased size of the dendritic block. This observation was consistent with the model proposed for amphiphiles with a packing parameter between 1/3 and 1/2, for which the geometry of the molecules favored the formation of cylindrical micelles. The sizes of these cylindrical aggregates were more uniform for the PEG 5000 derivative PEG 5000-G_2_(C17)_4_ than for PEG 2000-G_2_(C17)_4_, which had a broader diameter distribution. Moreover, for PEG 5000-G_2_(C17)_4_ the aggregates appeared to be strictly cylindrical, but for PEG 2000-G_2_(C17)_4_ some spherical aggregates were also observed.

For compounds PEG 2000-G_3_(C17)_8_ and PEG 5000-G_3_(C17)_8_, the interactions between the lipophilic blocks of the macromolecules gave rise to elongated self-assembled architectures with a mean thickness between 6 and 10 nm (Figure 3). However, the difference between the architectures of their corresponding aggregates was more significant than for the pairs of compounds discussed above. Whereas PEG 5000-G_3_(C17)_8_ formed elongated tubular aggregates, PEG 2000-G_3_(C17)_8_ provided rod-like structures that were reminiscent of flexible bilayers forming connected multilamellar aggregates. These experimental results suggested that the PEG 5000 derivative could be included within the group of amphiphiles with a packing parameter between 1/3 and 1/2, which self-aggregated into cylindrical micelles, while the PEG 2000 derivative corresponded to a packing parameter between 1/2 and 1, characteristic of amphiphiles with a lower hydrophilic content.

If one considered compounds formed by the same length of hydrophilic polymeric chain and different generations of lipophilic dendron, the trend suggested by the packing parameter models could be confirmed again. Comparison of the images of the PEG 2000 derivatives of the three generations or those of the PEG 5000 derivatives showed the transition from spherical micelles (*p* < 1/3) to more elongated micelles (1/3 < *p* < 1/2), to long tubular micelles or flexible bilayers (1/3 < *p* < 1) (Figure 3) for both series.

### 3.3. Encapsulation Ability

In light of the formation of micellar aggregates in water, studies were carried out to evaluate the possibility of forming aggregate/drug complexes with plitidepsin, a lipophilic anticancer drug. This drug was originally isolated from the Mediterranean tunicate *Aplidium albicans*, and is currently produced by chemical synthesis (PharmaMar S.A., Madrid, Spain) [42,43]. Plitidepsin is currently in phase II clinical trials for solid and hematological malignant neoplasias-like T cell lymphoma and in phase III clinical trials for multiple myeloma [44,45]. It provokes cell apoptosis by triggering mitochondrial cytochrome c release. Nevertheless, this drug has extremely low solubility in aqueous media, and its administration requires surfactants to increase the drug’s half-life in the bloodstream and to improve its bioavailability [46].

A procedure based on the oil-in-water method was performed. Increasing the lipophilic nature of aggregates upon addition of plitidepsin led to the precipitation of a certain amount of the amphiphilic HLDBC together with excess plitidepsin. The amount of drug that remained in solution was calculated by HPLC and the resulting value allowed the calculation of the actual concentration of HLDBC after drug encapsulation (Table 1 and Section S3). As a general trend, all of the compounds showed good properties in terms of forming host–guest systems, since they were able to encapsulate from 5% (0.056 mM, 0.062 mg/mL) to 100% of the plitidepsin in solution (1 mM, 1.11 mg/mL). Indeed, the lipophilic dendritic block provided a suitable environment for the hydrophobic drug, whereas the PEG block promoted stabilization of the whole system in water.

PEG 5000-G_3_(C17)_8_ gave the best results in this series since it can encapsulate all of the plitidepsin added (1 mM, 1.11 mg/mL). For the rest of the polymers, the amount of encapsulated plitidepsin was lower, and this reduction was more significant for HLDBCs with the smallest lipophilic dendritic block, i.e., the G_1_(C17)_2_ dendron. This finding confirmed the importance of the size of the lipophilic dendritic block, which was able to create a favorable environment for the stabilization of a lipophilic drug. Furthermore, the importance of the balance between the lipophilic and the hydrophilic content was also clear on considering the results obtained for compounds PEG 2000-G_3_(C17)_8_ and PEG 5000-G_3_(C17)_8_. The former compound had the highest lipophilic content (Lc = 39%), whereas the latter compound (Lc = 24%) was able to encapsulate and stabilize in water the highest quantity of drug due to its larger hydrophilic portion. In general, these compounds provided an advantageous combination of a high generation lipophilic dendron, which favored encapsulation of the lipophilic drug by hydrophobic interactions with the alkyl chains, and the high molecular weight of the hydrophilic PEG block, which helped to solubilize the entire system in water.

### 3.4. In Vitro Antitumoral Activity

The in vitro antitumoral activity of systems that contained plitidepsin at levels higher than 0.2 mg/mL and a drug/HLDBC ratio higher than 0.1, i.e., PEG 2000-G_2_(C17)_4_, PEG 2000-G_3_(C17)_8_, PEG 5000-G_2_(C17)_4_, and PEG 5000-G_3_(C17)_8_ were studied. The cytotoxicity of these systems was estimated on four human cancer cell-lines (A549 lung-NSCLC, HT29 colon, MDA-MB-231 breast, and PSN1 pancreas). The first three cancer cell-lines correspond to cancer with the highest prevalence while the fourth one corresponds to a cancer with a poor clinical outcome, and this makes them relevant enough to be tested for novel antitumoral agents with therapeutic potential. Three dose-response parameters were determined for each experimental agent: GI50 (compound concentration that produced 50% inhibition in cell growth compared to control cells); TGI (compound concentration that produced total growth inhibition compared to control cells); and LC50 (compound concentration that produced 50% net cell death).

Prior to testing the antitumoral activity of the host–guest systems, the biocompatibility of the amphiphiles was evaluated in the same four cancer cell-lines. HLDBCs alone did not show cytotoxic activity against the tumor cells even at the maximum concentration tested, 10 μg/mL. This finding verified that the cytotoxicity observed for the host–guest complexes was not caused by the HLDBC derivatives but by the encapsulated plitidepsin. Moreover, as plitidepsin has a very high activity against certain cancer cells (the order of magnitude is 10^−9^ M for NSCLC lung cancer cells, HT29 colon, breast, or pancreas cancer cells), it is important to retain this toxicity in any drug formulation.

**Figure 4:**
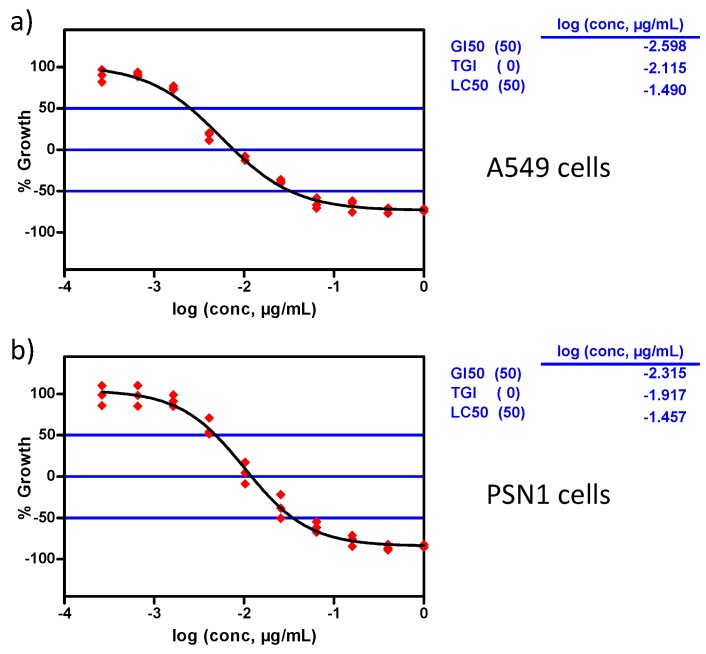
Dose-response (DR) curves for the complex 5000-G_3_(C17)_8_/plitidepsin representing cell growth percentage versus plitidepsin concentration measured in (**a**) lung-NSCLC (A549) and (**b**) pancreas (PSN1) tumoral cell-lines (GI50, 50% cell growth inhibition; TGI, total growth inhibition; LC50, 50% net cell death).

The HLDBC–plitidepsin complexes were tested in dose-response (DR) curves from 10^−1^ to 2.6·10^−5^ μg/mL. As representative examples, the DR curves corresponding to the PEG 5000-G_3_(C17)_8_/plitidepsin complex in lung-NSCLC (A549) and pancreas (PSN1) tumoral cell-lines are represented in Figure 4. GI50, TGI, and LC50 are calculated for the four plitidepsin host–guest complexes, and the results are gathered in the Table 2. Regardless of the HLDBC carrier, all four carrier–drug complexes showed cytotoxic activity in the low nanomolar range, with GI50 values in the range from 1.9 to 5.4 ng/mL, which was similar to that of plitidepsin itself (included as a positive control) and ensured limited side effects. These results indicated that the natural anti-cancer activity of the drug was not altered by the encapsulation process and that these HLDBCs provided a suitable alternative to the stabilizing surfactants currently employed in plitidepsin formulations [47] or other previously developed nanocapsules based on poly-amino acid–PEG derivatives [46].

## 4. Conclusions

Six amphiphilic hybrid linear-dendritic block copolymers (HLDBCs) have been studied. Due to their amphiphilic nature, all of the compounds self-assembled in water to form supramolecular micellar architectures that ranged from spherical to cylindrical to flexible bilayer. The architecture formed depended on the size ratio between the hydrophilic and lipophilic fractions. The aggregates proved to be useful as host systems to encapsulate a hydrophobic drug, namely plitidepsin, and the best amphiphilic compound in this respect was PEG 5000-G_3_(C17)_8_. The results demonstrated the importance of the size of the lipophilic fraction and the influence of the balance between the lipophilic and the hydrophilic content in order to stabilize in water the highest quantity of the drug. The appropriate combination of a high generation lipophilic dendritic block, which favors encapsulation of the lipophilic drug, and a high molecular weight hydrophilic linear PEG block, which helps to solubilize the entire system in water, is the key factor that makes these systems suitable as drug carriers. The effectiveness of these plitidepsin-loaded systems was studied in four cancer cell-lines and the results showed that the natural anti-cancer activity of the drug was not altered by the encapsulation process. The HLDBCs remained totally biocompatible at the concentrations used for encapsulation.

## Figures and Tables

**Table 1:** Lipophilic content (Lc) of HLDBC derivatives, critical micelle concentration (CMC) values, and drug and HLDBC concentrations after drug encapsulation, loading capacity (mg plitidepsin/mg HLDBC), and encapsulation efficiency (EE).

HLDBC	Lc ^a^ (%)	CMC		Plitidepsin (mg/mL)	HLDBC ^b^ (mg/mL)	Loading Capacity	EE (%)
		(mg/mL)	(mol/L)				
PEG 2000-G_1_(C17)_2_	18	0.0081	3.06∙10^−6^	0.062	1.1	0.056	5.6
PEG 2000-G_2_(C17)_4_	28	0.0048	1.40∙10^−6^	0.224	1.1	0.204	20
PEG 2000-G_3_(C17)_8_	39	0.0117	2.37∙10^−6^	0.207	1.3	0.159	19
PEG 5000-G_1_(C17)_2_	8	0.0017	1.73∙10^−6^	0.155	2.1	0.074	14
PEG 5000-G_2_(C17)_4_	15	0.0016	1.56∙10^−6^	0.207	2.1	0.100	19
PEG 5000-G_3_(C17)_8_	24	0.0018	1.76∙10^−6^	1.110	3.2	0.347	100

^a^ The lipophilic content represents the weight percentage of lipophilic portion present in the molecule (–(CH_2_)_16_CH_3_ of the stearic acid chains). ^b^ HLDBC concentration in water was calculated by subtracting the mass of HLDBC that precipitated during the encapsulation process from the initial mass of HLDBC (see Section S3).

**Table 2:** Cytotoxicity data for plitidepsin alone and the drug encapsulated in the four selected HLDBCs.

	GI50	TGI	LC50
	(ng/mL)	(ng/mL)	(ng/mL)
		**Plitidepsin**	
**Lung-NSCLC (A549)**	2.8	7.8	28.0
**Colon (HT29)**	2.4	6.7	>100
**Breast (MDA-MB-231)**	3.9	11.0	32.0
**Pancreas (PSN1)**	4.3	10.0	31.0
		**PEG 2000-G_2_(C17)_4_**	
**Lung-NSCLC (A549)**	3.2	9.4	40.0
**Colon (HT29)**	2.6	15.0	>1000
**Breast (MDA-MB-231)**	4.9	14.0	46.0
**Pancreas (PSN1)**	4.1	12.0	44.0
		**PEG 2000-G_3_(C17)_8_**	
**Lung-NSCLC (A549)**	1.9	8.1	50.0
**Colon (HT29)**	1.6	17.0	>1000
**Breast (MDA-MB-231)**	3.6	12.0	42.0
**Pancreas (PSN1)**	2.8	8.9	31.0
		**PEG 5000-G_2_(C17)_4_**	
**Lung-NSCLC (A549)**	3.1	9.5	41.0
**Colon (HT29)**	2.8	13.0	>1000
**Breast (MDA-MB-231)**	5.4	15.0	47.0
**Pancreas (PSN1)**	4.5	13.0	38.0
		**PEG 5000-G_3_(C17)_8_**	
**Lung-NSCLC (A549)**	2.5	7.7	32.0
**Colon (HT29)**	2.1	11.0	>1000
**Breast (MDA-MB-231)**	4.3	13.0	43.0
**Pancreas (PSN1)**	4.8	12.0	35.0

## Supplementary Materials

The Supplementary Materials are available online at http://www.mdpi.com/2079-4991/9/2/161/s1, chemical characterization of HLDBCs, aggregate formation, and plitidepsin encapsulation.

## Abbreviations

^1^H-NMR, 1-proton nuclear magnetic resonance. ^13^C-NMR, 13-carbon nuclear magnetic resonance. A549 cells, lung, non-small cell lung cancer cells. ANCI, American National Cancer Institute. Bis-MPA, 2,2′-bis(hydroxymethyl)propionic acid. CMC, critical micellar concentration. CuAAC, Cu(I)-catalyzed alkyne-azide cycloaddition. Đ, polydispersity index. DCC, *N*,*N*′-dicyclohexylcarbodiimide. DMEM, Dulbecco’s modified eagle medium. DMF, dimethylformamide. DMPA, 4-dimethyl aminopyridine. DMSO, dimethyl sulfoxide. DPTS, 4-(dimethylamno)pyridinium *p*-toluenesulfonate. DR, dose-response. EE, encapsulation efficiency. FBS, fetal bovine serum. FTIR, Fourier transform infrared spectroscopy. GI50, compound concentration that produces 50% cell growth inhibition as compared to cultures. HLDBC, hybrid linear-dendritic block copolymers. HPLC, high pressure liquid chromatography. HT29 cells, colon cancer cells. Lc, lipophilic content. LC50, compound concentration that produces 50% net cell death cytotoxic effect. MALDI-TOF, matrix assisted laser desorption/ionization–time of flight. MDA-MB-231 cells, breast cancerous cells. *M*_n_, number average molecular weight. MPS, mononuclear phagocytic system. MW, molecular weight. PEG, poly(ethylene glycol). PSN1 cells, pancreas cancerous cells. TEM, transmission electron microscopy. TGI, compound concentration that causes total cell growth inhibition, i.e., cytostatic effect, as compared to cell cultures.
