# Supplementary material for: Self-Assembling Hybrid Linear-Dendritic Block Copolymers: The Design of Nano-Carriers for Lipophilic Antitumoral Drugs
Source: Nanomaterials (Basel). 2019 Jan 29;9(2):161. doi: 10.3390/nano9020161 (PMC6409548; doi:10.3390/nano9020161)
Supplement: Supplementary file 1 [file nanomaterials-09-00161-s001.pdf]

SUPPORTING INFORMATION

Self-assembling hybrid linear dendritic block copolymers: designing nano-carriers for lipophilic antitumoral drugs

Elisabetta Fedeli, Alexandre Lancelot, Juan Manuel Domínguez, José Luis Serrano, Pilar Calvo,* Teresa Sierra*

S1 - Chemical characterization of the HLDBC derivatives, p 2

S2- Determination of the Critical Micellar Concentration (CMC), p 5

S3- Encapsulation of plitidepsin, p 6

S1 - Chemical characterization of the HLDBC derivatives.

^{1}H-NMR

Figure S1 gathers a detail of the ^{1}H-NMR spectra of the starting materials and the final compound (**PEG 5000-G_3(C17)$_8$**). The peak relative to the "**b**" protons ($-CH_2-$) in α position with respect to the triazole ring on the side of the lipophilic dendron appears downfield shifted at δ_H=5.24 ppm, while in the starting lipophilic dendron it is recorded at δ_H=4.73 ppm. Another meaningful result is the downfield shift of the triplet at δ_H=4.54 ppm ("**c**" protons) relative to the methylene (CH_2-$\underline{CH_2}$-N-N=N-) in α to the triazole ring. The same signal in the starting poly(ethylene glycol)methyl ether azide is recorded at δ_H=3.86 ppm. Also the triplet related to "**d**" protons, that in the starting material is not visible as it appears together with the multiplet of the CH_2 of the PEG, appears at δ_H=3.88 ppm.

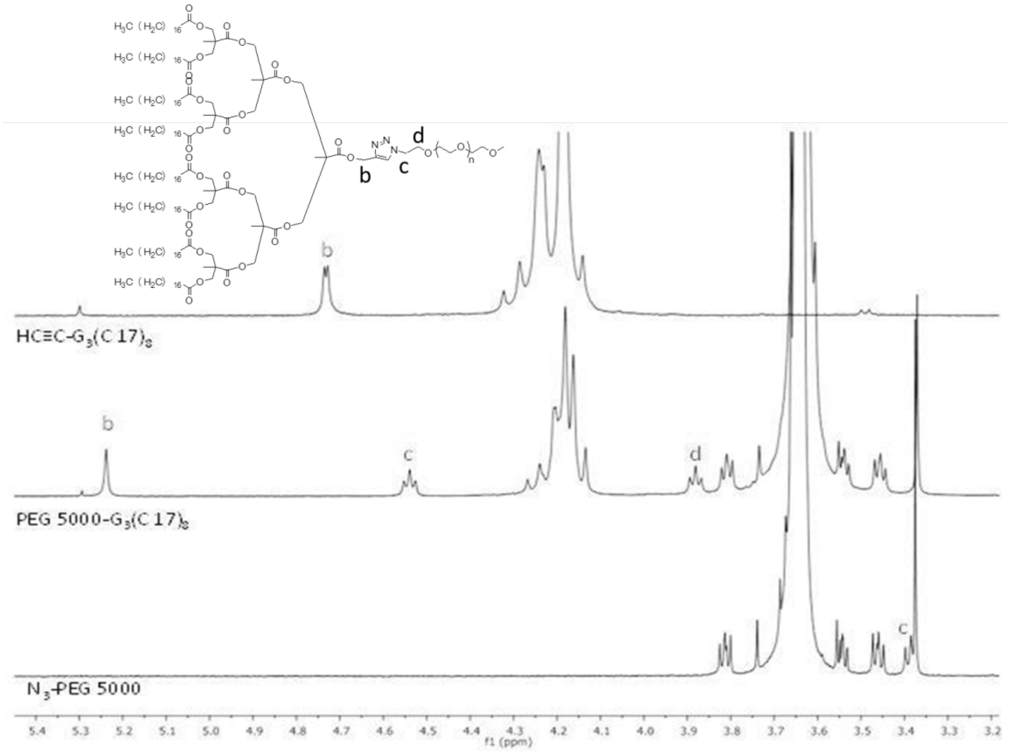

Figure S1: Comparison between the ^{1}H-NMR spectra of compound **PEG 5000-G_3(C17)$_8$** and starting reagents. Detail of the shifting of the most significant signals.

FTIR

The FTIR was a useful analysis method to check the success of the reaction. In fact, neither the characteristic peak of the azide group (2100 cm^{-1}) nor the one corresponding to the stretching vibration of the H-C bond of the alkyne group (3300 cm^{-1}) were visible in the spectrum of the final compounds. As an example, the FTIR spectrum of compound PEG 2000-$G_2(C17)_4$ is compared to the FTIR spectra of the starting products (figure S2).

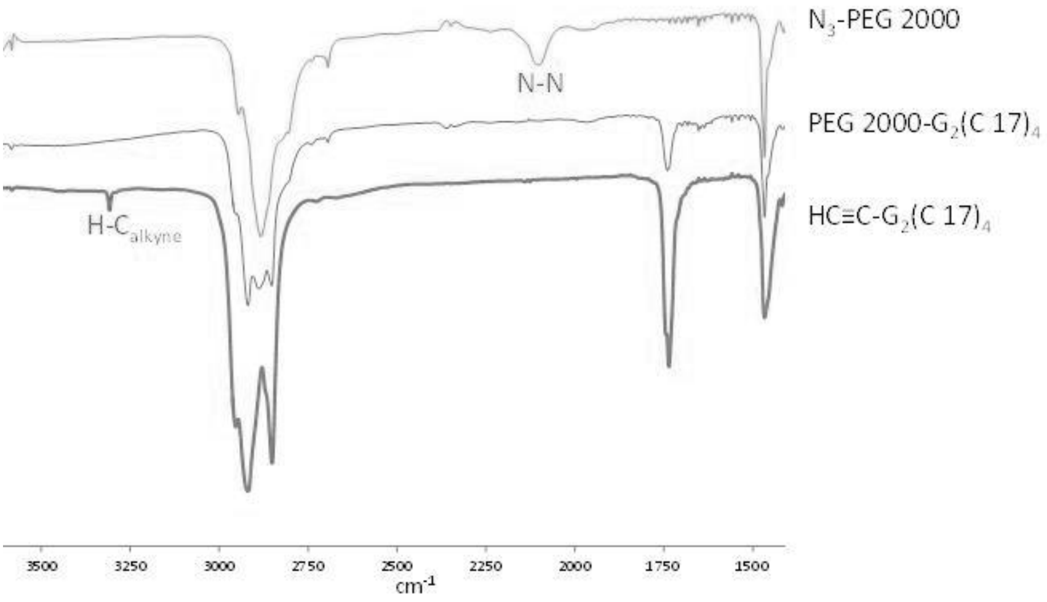

Figure S2: Comparison between the FTIR spectra of compounds **PEG 2000-$G_2(C17)_4$** (central), and its corresponding starting reagents.

GPC and MS

The chromatogram of compound **PEG 5000-G_2(C17)$_4$** and the chromatograms of its two starting reagents, compound **HC≡C-G_2(C17)$_4$** and poly(ethylene glycol)methyl ether azide of 5000 Mn (**N_3-PEG 5000**), are gathered in figure S3. The asymmetry of the peak of the final dendrimeric derivative can be due to several factors as, for instance, the polydispersity of the starting PEG, the interaction between the silica-based packing of the column and the product, or the self-association of molecules giving species that interact differently with the chromatographic column. As expected, the retention time of the final products is lower with respect to the ones of the starting materials due to the increased hydrodynamic volume that provokes a more rapid passage of the product through the column. The trend of the retention times is the same for all the samples, being the most retained product the lipophilic dendron, with the lowest hydrodynamic volume, followed by the starting poly(ethylene glycol)methyl ether azide and then by the final HLDBC derivative.

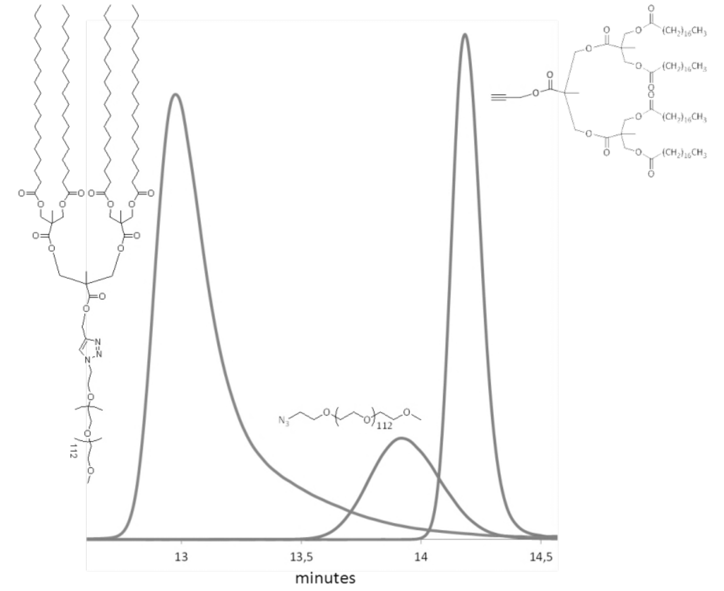

Figure S3: Comparison between the chromatogram of the final product **PEG 5000-G_2(C17)$_4$** and the chromatograms of the two starting reagents.

Table S1 gathers the data calculated from the GPC analyses performed for each final product; the molecular weight is calculated by summing up the atomic masses of all the atoms that constitute the structure.

Table S1: Results of GPC and MS analyses.

		GPC		MALDI-TOF
**HLDBC**	**MM[a]**	**M_n[b]**	**Đ[b]**	**M_n[c]**
PEG2000-derivatives				
PEG2000-G_1(C17)$_2$	2649	4084	1.04	2317
PEG2000-G_2(C17)$_4$	3414	5665	1.00	3325
PEG2000-G_3(C17)$_8$	4944	4015	1.05	4902
PEG5000-derivatives				
PEG5000-G_1(C17)$_2$	5649	6380	1.05	5400
PEG5000-G_2(C17)$_4$	6414	6721	1.04	6202
PEG5000-G_3(C17)$_8$	7944	5944	1.08	7831

[a] molecular mass calculated by summing up the atomic masses of all the atoms of the structure. [b] number average molecular mass obtained by GPC and polydispersity index; both the indexes were calculated using PMMA as matrix and using THF as solvent. [c] number average molecular mass obtained by mass spectrometry using PolyTool 1.15 of the software Bruker Daltonics.

The M_n and Đ values obtained by GPC analysis were determined using PMMA standard; the different structure of the PMMA and the proposed molecules may be the cause of the difference between the experimental

data obtained from GPC (M_n) and the theoretical ones (MM). Observing the M_n values, it is possible to deduce a certain unexpected trend. In both series (PEG 2000 and PEG 5000 derivatives), the compound with the highest M_n is the second generation derivative, followed by the first generation one and, as the most retained product, the third generation derivative. This uncommon behavior could be due to self-aggregation of the amphiphilic molecules giving rise to more complex structures with higher hydrodynamic volumes that interact differently with the silica-based chromatographic column. This hypothesis could also justify the higher asymmetry of the peaks of the final products respect to those of the corresponding original PEGs that present a lower asymmetry.

Being the hybrid derivatives composed by monodispersed dendrons and polydispersed polymers, the mass spectra show a molecular mass distribution in which each peak is separated from the other by 44 that corresponded to the molecular mass of the repeating unit of the poly(ethylene glycol). Figure S4 represents the mass spectra of HLDBCs derivatives of PEG 2000: **PEG 2000-$G_1(C17)_2$**, **PEG 2000-$G_2(C17)_4$**, **PEG 2000-$G_3(C17)_8$**. An increase of the molecular mass due to the change of the generation linked to the PEG derivative is clearly observed; the original spectrum of the commercial polymer used in the reaction is also included.

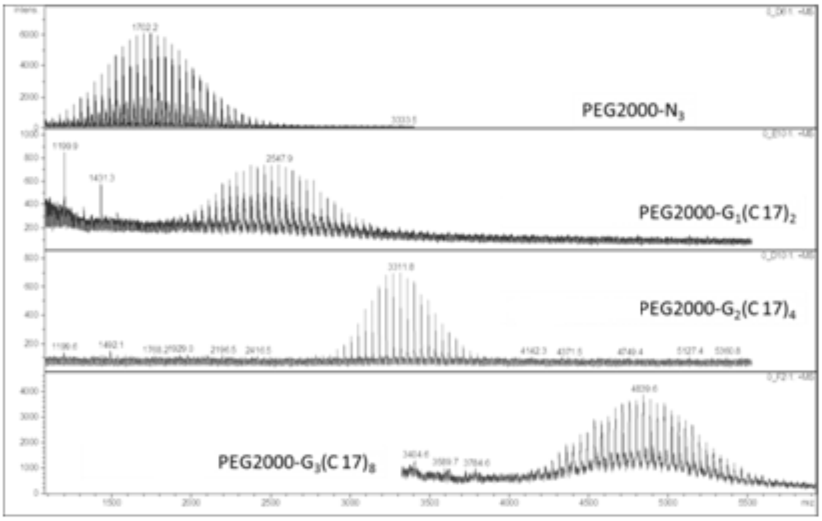

Figure S4: MALTI-TOF Mass spectra corresponding to HLDBCs composed by PEG 2000 and dendritic lipophilic dendrons of first, second and third generation. The mass spectrum of the commercial poly(ethylene glycol)methyl ether azide used for the reactions is also included.

All the spectra show that every peak distribution is almost symmetrical and similar in width to the one of the starting polymer (N_3-PEG 2000). M_n and Đ values (table S1) were estimated using a special tool of the software employed to process the mass spectra, i.e. PolyTool 1.15 of the software Bruker Daltonics. Compared to the values obtained by GPC analysis, which uses molecular weight distribution standards (MWD), the ones estimated by PolyTool are considered more reliable as the software allows the specification of parameters of the structure, such as ending groups, the MM of the repeating units, and compares the data with an internal database, looking for the best fitting match.

S2- Determination of the Critical Micellar Concentration (CMC):

Pyrene fluorescence excitation method was employed to determine the CMC of the HLDBC derivatives. It is based on the fact that the excitation spectrum of pyrene dissolved in water shows a band at 332 nm; when pyrene is dissolved in a hydrophobic media, the maximum of the band shifts to 335 nm. When an amphiphile forms micellar aggregates in water, pyrene, being hydrophobic, progressively migrates to the hydrophobic inner core of the micelles. Its migration produces the shift of the excitation band from 332 nm towards 335 nm, since pyrene passes from a polar environment (water) to a non-polar environment (core of the micelles).

The I_{335}/I_{332} ratio of water solutions of each amphiphilic compound containing pyrene ($6 \cdot 10^{-8}$ mol/L) was measured for concentrations of the amphiphile from 0.0001 mg/mL to 1mg/mL. Each solution was prepared by dissolving the corresponding amount of compound in dichloromethane, adding 1 mL of milli-Q water and evaporating the dichloromethane to open air at room temperature. Once the organic solvent was completely removed, 5 μL of a solution of pyrene with concentration $12 \cdot 10^{-6}$ mol/L was added. The pyrene solution was prepared dissolving 0.485 mg of pyrene in 500 μL of acetone; the solution was added to distilled water using a volumetric flask of 200 mL. The organic solvent was evaporated under reduced pressure for 4 hours. The minimum variation of concentration due to the volume occupied by the dissolved pyrene was not considered affecting the final concentration in solution. The solutions containing the samples and pyrene were stirred for 4 hours at room temperature to allow the formation of the micelles, the complete encapsulation of the probe and the stabilization of the systems.

Due to the different nature of the compounds, not all the products were soluble at high concentrations. In these cases, the solutions that presented solid precipitate were discarded and the analysis was performed just in the completely soluble samples.

S3 - Encapsulation of plitidepsin

General Procedure

The ability of the amphiphilic compounds to encapsulate a hydrophobic guest was evaluated using plitidepsin as lipophilic drug. The drug was stored at -30°C and used without further purifications.

The encapsulation procedure was performed employing the "oil-in-water method" adapted for this specific procedure.

For the initial concentration of the amphiphiles, the highest used to establish the maximum solubility limit: 0.5 μmol/mL was maintained.

The following procedure was established to encapsulate plitidepsin:

- The amphiphile and Plitidepsin were dissolved in separated vials in 0.5 mL of dichloromethane. The quantity of each component was chosen in order to obtain 1 mL of water solution with concentration 0.5 μmol/ml of the dendrimeric derivative and 1 μmol/mL of plitidepsin (1.11 mg, M_w 1110.34g/mol).
- The two organic solutions were mixed together and stirred for few minutes in a closed vial to allow the complete stabilization of the solution.
- 1 mL of Milli-Q water was gently added and the open vial was shaken in an orbital shaker at room temperature till complete evaporation of the organic solvent.

Determination of the concentrations in HLDBC derivatives and Plitidepsin in water:

After the complete evaporation of the organic fraction, a solid precipitate appeared. The exact concentration of plitidepsin remained in solution was established by HPLC using a standard procedure developed by PharmaMar. In order to establish the real concentration in solution of the amphiphile, the water solution of the host-guest complex was washed twice with dichloromethane to dissolve the solid precipitate. The organic fraction was removed and put in a new vial with known weight; this vial was dried under vacuum for 3 days at 50°C until its weight remained constant. By weight difference, the quantity of precipitate was calculated.

Since the concentration of plitidepsin in solution was established by HPLC, its amount in the solid precipitate was calculated by difference with the initial amount of plitidepsin (1.11 mg). Knowing the global amount of the precipitate and the fraction of plitidepsin, the quantity of amphiphile in the precipitate and, consequently its concentration in water, were extrapolated.
